# Association of American Medical Editors

**Published:** 1871-06

**Authors:** 


### Proceedings of Societies.

### ASSOCIATION OF AMERICAN MEDICAL EDITORS. THIRD ANNUAL MEETING.

At ten o'clock, May 1st, 1871, the Medical Journal Association met at the rooms of the San Francisco Medical Society, on Sutter Street. Dr. H. R. Storer, of Boston, President of the Association, called the meeting to order, and stated that from the arrangements being made, the Delegates of the Medical Association from the East would have a pleasant time.

In the absence of the Secretary, Dr. Henry Gibbons, Jr., was elected Secretary *pro tem.*

The proceedings of the last annual meeting not being at hand, Dr. N. S. Davis, of Chicago, read the plan of organization of the Society, the object of which is to cultivate fraternal relations among members of the medical profession, to urge a higher standard of preliminary education of persons proposing to enter the profession, and to collate vital statistics.

AMENDMENT TO THE CONSTITUTION.

Dr. N. S. Davis, from the Committee on Revision of Laws, presented the following amendment to the constitution relating to officers, which, on motion, was adopted:—

"The officers of the Association shall be a President, Vice-President, Secretary, Assistant-Secretary, and Treasurer. All except the Assistant-Secretary to be elected annually by ballot, and shall commence their term of service at the opening of the next annual meeting after their election. The Assistant-Secretary shall be appointed annually by the President, and shall be resident at the place of the next succeeding meeting.

"It shall be the duty of the Secretary to procure a suitable book, and keep in it a permanent record of the organization, list of members, and proceedings of the Association, and, in case of his inability to attend any particular meeting, he shall, prior to such meeting, transmit the book of records to the Assistant-Secretary.

"It shall be the duty of the Assistant-Secretary to procure suitable accommodations for the annual meetings of the Association, and to assist the Secretary in the performance of his duties."

JOURNALS REPRESENTED.

The following journals were represented at the meeting:

*Journal of the Gynecological Society of Boston*, Dr. H. R. Storer; Chicago *Medical Examiner*, Dr. Davis; *American Practitioner*, Dr. Yandell; *National Medical Journal*, Dr. Toner; *Pacific Medical Journal*, Dr. Henry Gibbons.

On motion, the Committee on Registration was discontinued, as they had presented no report in three years.

On motion, the Committee on Foreign Exchanges, consisting of Drs. Dawson, of New York; Parvin, of Louisville; and Mitchell, of New Orleans, was continued; and Dr. Jones, of New Orleans, was added to the Committee.

The President stated that he had communicated with Professor Henry, of the Smithsonian Institute, who had promised to aid in keeping up the International exchanges. The Chair also stated that last year the Society only numbered 12 or 13 journals, but at present numbered about 40 members.

RESOLUTIONS.

Dr. Davis offered the following resolutions:

*Resolved*, That the social, educational, and scientific interests of the profession would be greatly promoted by a more complete organization in every State and district in our counrty, such organization being calculated not only to direct and diffuse knowledge, but also to afford the most efficient means for procuring concerted and efficient action on all important questions of medical education and progress.

*Resolved*, That deficiency in the general education of young men entering upon the study of medicine in this country is an event of great magnitude, not only constituting a barrier to individual progress in professional life, but greatly lessening the general reputation and usefulness of the profession.

*Resolved*, That the members of this Association be requested to use their respective medical periodicals as agencies for calling the special attention of the profession to the topics mentioned in the foregoing resolutions, until such a professional sentiment is created that no regular practitioner will feel at liberty to receive a student into his office who does not present testimonials from some competent source that he has at least a competent knowledge of the ordinary branches of education, including the lower mathematics and the natural sciences; and the social organizations are so far complete that the several State Societies become the real and authoritative representatives of the profession in each State.

On motion, they were laid over until the evening session.

INVITATIONS.

The Chairman read an invitation he had received from Dr. Thomas M. Logan, inviting the Association to attend the meeting of the California State Medical Society.

Dr. Yandell moved that the invitation be accepted, and to reciprocate by inviting the California State Medical Society to attend the meeting of the Association, and address by Dr. Storer in the evening.

The meeting then adjourned till eight o'clock P.M.

---

EVENING SESSION.

Pursuant to adjournment, the Association met at eight o'clock in the evening, in the hall of the Young Men's Christian

Association, quite a large number of ladies and gentlemen being present.

Dr. Storer, the President, called the meeting to order, and delivered the annual address. The address was listened to with marked attention, and the speaker applauded at its close.

The Association then proceeded to business. Dr. Dawson, of the *American Journal of Obstetrics* of New York, was reported present.

ADOPTED.

The resolutions introduced at the morning session by Dr. Davis were taken from the table and read.

Dr. Yandell moved that the resolutions be adopted and published in all the medical journals. Carried.

ELECTION OF OFFICERS.

On motion of Dr. Davis, the Association proceeded to the election of officers for the ensuing year, with the following result: President, Dr. B. F. Dawson, of New York; Vice-President, Dr. H. Gibbons, Jr., of San Francisco; Secretary, Dr. F. H. Davis, of Chicago.

THANKS.

On motion of Dr. Yandell, the thanks of the Association were tendered to Dr. Toner in recognition of his labors in the preparation of an index of all the physicians of the country, and the contents of all medical journals since their publication, with a request that he continue the work, and report at the next annual meeting.

The Association then adjourned until May next.

